# Development and validation of the Cancer Dyspnoea Scale: a multidimensional, brief, self-rating scale

**DOI:** 10.1054/bjoc.1999.1002

**Published:** 2000-02

**Authors:** K Tanaka, T Akechi, T Okuyama, Y Nishiwaki, Y Uchitomi

**Affiliations:** Psycho-Oncology Division, National Cancer Center Research Institute East, Chiba, Japan; Divisions of Thoracic Oncology and Psychiatry, National Cancer Center, Hospital East, 6-5-1 Kashiwanoha, Kashiwa, Chiba, 277-8577, Japan; Psychiatry Division, National Cancer Center Hospital, Tokyo, Japan

**Keywords:** cancer, dyspnoea, assessment, scale, multidimensional, validation

## Abstract

Dyspnoea is one of the most frequent and refractory symptoms in cancer patients. Lack of an appropriate assessment tool for dyspnoea seems to disturb establishment of management strategy. The purpose of this study was to develop and validate a brief self-rating scale to assess the multidimensional nature of dyspnoea in cancer patients. We developed a 12-item scale, the Cancer Dyspnoea Scale (CDS), composed of three factors (sense of effort/sense of anxiety/sense of discomfort), by using factor analysis. One hundred and sixty-six patients with advanced or recurrent lung cancer participated in the validation phase. The CDS showed good feasibility (average time required to complete it was 140 s). Construct validity, confirmed by repeating factor analysis, was good. Convergent validity, confirmed by a relation to Visual Analogue Scale of dyspnoea and modified Borg's scale, was also good (average: *r* = 0.57 and 0.52, respectively, and both *P*< 0.001). The CDS had good internal consistency (average Cronbach's alpha = 0.86) and stability (average test-retest reliability *r* = 0.66, *P*< 0.005). The present study demonstrated that the CDS is a brief, valid and feasible scale for assessing the multidimensional nature of dyspnoea in cancer patients. © 2000 Cancer Research Campaign


*British Journal of Cancer* (2000) **82**(4), 800–805
© 2000 Cancer Research Campaign
DOI: 10.1054/ bjoc.1999.1002, available online at http://www.idealibrary.com on IDEAL®

### Development and validation of the Cancer Dyspnoea Scale: a multidimensional, brief, self-rating scale

**K Tanaka[1,2], T Akechi[1,4], T Okuyama[1,]*, Y Nishiwaki[2] and Y Uchitomi[1,3]**

[1]Psycho-Oncology Division, National Cancer Center Research Institute East, Chiba, Japan; Divisions of [2]Thoracic Oncology and [3]Psychiatry, National Cancer Center, Hospital East, 6-5-1 Kashiwanoha, Kashiwa, Chiba 277-8577, Japan; [4]Psychiatry Division, National Cancer Center Hospital, Tokyo, Japan

**Summary** Dyspnoea is one of the most frequent and refractory symptoms in cancer patients. Lack of an appropriate assessment tool for dyspnoea seems to disturb establishment of management strategy. The purpose of this study was to develop and validate a brief self-rating scale to assess the multidimensional nature of dyspnoea in cancer patients. We developed a 12-item scale, the Cancer Dyspnoea Scale (CDS), composed of three factors (sense of effort/sense of anxiety/sense of discomfort), by using factor analysis. One hundred and sixty-six patients with advanced or recurrent lung cancer participated in the validation phase. The CDS showed good feasibility (average time required to complete it was 140 s). Construct validity, confirmed by repeating factor analysis, was good. Convergent validity, confirmed by a relation to Visual Analogue Scale of dyspnoea and modified Borg's scale, was also good (average: $r = 0.57$ and 0.52, respectively, and both $P < 0.001$). The CDS had good internal consistency (average Cronbach's alpha = 0.86) and stability (average test-retest reliability $r = 0.66$, $P < 0.005$). The present study demonstrated that the CDS is a brief, valid and feasible scale for assessing the multidimensional nature of dyspnoea in cancer patients. © 2000 Cancer Research Campaign

**Keywords:** cancer; dyspnoea; assessment; scale; multidimensional; validation

Dyspnoea is defined as 'an uncomfortable sensation of breathing' (Manning et al, 1995). It should be distinguished from respiratory failure, which is defined as pulmonary dysfunction with hypoxia and/or hypercapnoea. Published prevalence rates for dyspnoea in cancer patients range from 29% to 74% in the terminal stage (Reuben et al, 1986; Doyle et al, 1998). It is one of the most refractory symptoms (Higginson et al, 1989) in the terminal stage, even when no tumour involvement is demonstrated in the lung (Bruera et al, 1998). In spite of its high prevalence, limited research is available on adequate assessment and management (Higginson et al, 1989).

The pathophysiological mechanisms of dyspnoea are poorly understood despite extensive research. Aetiology of dyspnoea can not be always explained pathophysiologically. Some modulators, such as psychological state, cultural background, environment and life experiences, are recently considered to amplify or decrease the intensity of the symptom perceived at the cortical level (Ripamonti et al, 1997). Some studies have shown significant correlations between dyspnoea and psychological status (Burns et al, 1969; Dales et al, 1989; Gift et al, 1990; Moody et al, 1990; McCord et al, 1992; O'Connor et al, 1996). Some other studies have shown that the different terms describing dyspnoea are associated with aetiology and various stimuli (Simon et al, 1989, 1990; Elliott et al, 1991). These findings suggested that dyspnoea includes several qualitatively distinct sensations that arise from different mechanisms (Manning et al, 1995).

It is hypothesized that there might be several aspects of dyspnoea; however, few studies about subtypes of dyspnoea in cancer patients have been done and an appropriate assessment tool for dyspnoea in this population has not been established. Available scales are not appropriate for understanding the aetiologies and establishing a therapeutic strategy for them. Some scales evaluating the intensity of dyspnoea subjectively, such as Borg's scale (Borg, 1970) and the Visual Analog Scale of dyspnoea (Atkin, 1969), are simple and widely used, but multidimensional assessment cannot be achieved with them. Some other scales, which objectively measure physical effort evoking dyspnoea, such as Hugh–Jones scale (Fletcher et al, 1959) and others (Medical Research Council Committee, 1965; American Thoracic Society, 1978; McGavin et al, 1978), are not feasible for patients whose activity is limited by other symptoms or disability. They are sometimes not useful because perceived dyspnoea has not always been found to be correlated with the results of exercise tests and respiratory function tests (Burdon et al, 1983; Stoller et al, 1986; Maler et al, 1987).

Development of a new measure is crucial to investigating the aetiology and establishing a therapeutic strategy for dyspnoea (Bruera et al, 1998). The scale should:

1. comprise multidimensional aspects
2. be self-rating, because dyspnoea is subjective
3. be easy and simple enough to be completed by patients troubled by dyspnoea

*Received 1 June 1999*
*Revised 21 September 1999*
*Accepted 20 October 1999*

*Correspondence to:* Y Uchitomi, Psycho-Oncology Division, National Cancer Center Research Institute East, 6-5-1 Kashiwanoha, Kashiwa, Chiba 277-8577, Japan

*T Okuyama is an awardee of a Research Resident Fellowship from the Foundation for the Promotion of Cancer Research.

4. be evaluated not by physical effort evoking dyspnoea, but by perceived dyspnoea itself so that even bedridden patients can complete it
5. have its reliability and validity in cancer patients confirmed,
6. be sensitive to clinical changes due to treatment or progression of the disease over time.

The purpose of this study was: (1) to develop a brief self-rating scale to assess dyspnoea in cancer patients and (2) to validate it. We paid particular attention to the multidimensionality of dyspnoea, with the hypothesis that there might be psychological aspects as well as physiological ones, as some reports have suggested (Burns et al, 1969; Dales et al, 1989; Gift et al, 1990; Moody et al, 1990; McCord et al, 1992; O'Connor et al, 1996).

### METHODS

### Subjects

Cancer patients at the National Cancer Center Hospital East, Japan, participated. Eligible patients were required: (a) to have been pathologically diagnosed as having cancer and to have been informed of their diagnosis, (b) to be 18 years or older, (c) to be well enough to complete the questionnaire, (d) to not be suffering from severe mental or cognitive disorders. The study was approved by the Institutional Review Board and the Ethics Committee of the National Cancer Center. Written consent was obtained after each patient had been fully informed of the purpose of the study.

### Study design

The study consisted of two phases: (1) a development phase (to develop the dyspnoea scale) and (2) a validation phase (to confirm its feasibility, reliability and validity).

#### *Development phase*

First, terms which describe, represent and evaluate dyspnoea were collected in the following ways: (a) by interviewing dyspnoeic cancer patients closely in a clinical setting, (b) by brainstorming with medical experts (i.e. oncologists, psycho-oncologists and nurses engaged in thoracic oncology and in palliative care unit for more than 3 years) and (c) by picking up from reported papers on dyspnoea. After collecting a huge pool of terms, the medical experts made a majority decision after series of discussions to omit the terms that may: (a) be difficult for anyone to understand, that is, local dialect, jargon and vague vocabulary; (b) overlap each other, that is, linguistically synonym; and (c) be confounded with symptoms other than dyspnoea, for example, description of cough and sputum. A preliminary questionnaire with 5-point scale, ranging from 1 (not at all) to 5 (very much) was prepared.

Cancer patients were then asked to fill out this draft scale along with the Visual Analogue Scale (VAS) of dyspnoea and modified Borg's scale. Inappropriate items that met the following criteria were then eliminated from the draft scale: (a) items which quite a few patients required further explanation to complete, (b) items whose correlation with VAS of dyspnoea was not significant, and (c) items whose standard deviation of response was less than 1.0. These remaining items were then factor analysed by principal component analysis with varimax rotation. The number of factors were determined by the Scree test (Catell, 1978). Items that loaded less than 0.65 were deleted from each subscale.

Translation of the scale into an English version was completed by employing the standard 'forward–backward' translation procedure (Bonomi et al, 1996), which consists of the following steps:

1. two professional native English translators performed independent forward translations
2. a third, independent translator resolved discrepancies
3. the fourth, independent professional translator, a native Japanese, back translated the reconciled version
4. three bilingual experts reviewed the revised version and decided on the final version.

#### *Validation phase*

Additional eligibility criteria were applied in this phase, so that the influence of confounding factors in a heterogeneous sample could be avoided. Participants were diagnosed as having lung cancer in an advanced stage (i.e. in clinical stage IIIa [unresectable], IIIb, or IV) or recurrent stage. Consecutive outpatients and cross-sectional inpatients in the Thoracic Oncology Division were asked to complete the Cancer Dyspnoea Scale (CDS), after brief instruction. In addition to this scale, outpatients were requested to complete other measures at home on the hospital-visit day and to mail it by the following day. If there were any blanks, telephone inquiry was made to obtain the missing answers, as agreed with the participants. Participants were given a 500-yen prepaid telephone-card for participating in the study.

### Measures

#### *Modified Borg's scale*

Modified Borg's scale is a 12-point numerical plus verbal scale that is easy to administer, is reproducible and has been found to correlate with physiological parameters of lung disease in exercise trials (Wolkove et al, 1989; Mador et al, 1995).

#### *VAS of dyspnoea*

VAS of dyspnoea is a 100-mm line anchored by the terms 'no dyspnoea' and 'worst possible dyspnoea', on which intensity of dyspnoea is marked. It has also been validated (Gift et al, 1989), and is more sensitive and precise than Borg's scale (Muza et al, 1990).

#### *State-Trait Anxiety Inventory (STAI)*

The STAI was used to investigate associations between anxiety and the CDS. It consists of a 40-item self-rating questionnaire, evaluating state- and trait-anxiety separately (Spielberger et al, 1970). The Japanese version has also been validated (Nakazato et al, 1982).

#### *Physician's assessment, vital signs and laboratory data*

Performance Status (PS) defined by the Eastern Cooperative Oncology Group (ECOG) and the presence of pathophysiological causes of dyspnoea was clinically evaluated on the same day by physicians engaged in thoracic oncology for over 5 years. After sitting at rest for 5 min, the patient's oxygen saturation ($SpO_2$) was measured with a pulse oximeter at the digit.

### Feasibility

Inpatients were observed to see whether they had any difficulty in completing the CDS, and after they completed it they were asked

© 2000 Cancer Research Campaign 

directly if they had any difficulty in completing it. The time required for inpatients to complete it was measured.

### Validity

Construct validity (i.e. whether each subscale represents and correlates with each dimension) was evaluated by factor analysis followed by varimax rotation.

Intersubscale correlation (i.e. the strength of the correlations between subscales) was evaluated by calculating Pearson's correlations.

Convergent validity (i.e. the strength of the correlations between the subscale and aggregate, and other validated measures of dyspnoea) was assessed by Pearson's correlations with VAS of dyspnoea completed at the same time.

### Reliability

Internal consistency (i.e. homogeneity) of the multiple item scales was evaluated by calculating Cronbach's alpha coefficient.

Test–retest reliability (i.e. reproducibility) was evaluated in a group of consecutive outpatients who were asked to complete the scale twice, about a week apart, and mail it each time. Patients whose treatment, including all medications, was changed and/or who experienced any noteworthy clinical event during that period were excluded. The results on the two occasions were assessed by Pearson's correlation.

All statistical procedures were performed using SPSS 7.5.1J for Windows (SPSS Inc., 1997).

**Table 1** Demographic and clinical characteristics ($n$ = 166)

| Characteristics | | No. of patients (%) |
|---|---|---|
| Age (years) | Median | 64 |
| | Range | 27–87 |
| Sex | Male | 123 (74.1) |
| Married | | 147 (88.6) |
| Work outside the home | | 25 (16.1) |
| Education level (years) | | |
| | –9 (junior high school or less) | 90 (54.2) |
| | 10–12 (high school or less) | 51 (30.7) |
| | 13– (beyond high school) | 25 (15.1) |
| Outpatient | 135 (81.3) | |
| Histological type | Adenocarcinoma | 85 (51.2) |
| | Small-cell | 39 (23.5) |
| | Squamous cell | 38 (22.9) |
| | Others | 4 (2.4) |
| Clinical stage | | |
| No prior treatment | IIIa | 23 (13.9) |
| | IIIb | 56 (33.7) |
| | IV | 49 (29.5) |
| Recurrent case | | 38 (22.9) |
| Treatment | Surgery | 30 (18.1) |
| (multiple choice) | Chemotherapy | 129 (77.7) |
| | Radiotherapy | 69 (41.6) |
| | Pleurodesis | 8 (4.8) |
| PS (ECOG)* | 0 | 31 (18.7) |
| | 1 | 125 (75.3) |
| | 2 | 4 (2.4) |
| | 3 | 4 (2.4) |
| | 4 | 2 (1.2) |
| Days after diagnosis | Median | 273 |
| of cancer | Range | 14–3138 |

*Performance Status defined by Eastern Cooperative Oncology Group.

### RESULTS

### Development phase

Interviews with about 20 dyspnoeic inpatients in the Thoracic Oncology Division and Palliative Care Unit were held by the first author. Brainstorming was repeated by 11 oncologists, six psycho-oncologists and six nurses in the Thoracic Oncology Division and Palliative Care Unit. With these procedures, 179 terms were listed; most came from brainstorming and the remaining from interview and checking reviews. These terms were reduced according to the criteria described before. A preliminary questionnaire consisting of 24 items was then prepared and delivered to 117 cancer patients. There were more males (66.7%) than females, and the median age was 61 years (range 36–80 years). The most frequent cancer site was the lung (76.1%), followed by the breast (12.0%) and the oesophagus (5.1%). Approximately half of patients' cancers (49.6%) were in the advanced stage (clinical stage III–IV) or in the recurrent stage, and most of them (87.2%) had an ECOG PS of 0 or 1.

Factor analysis was performed on the remaining items, after discarding six inappropriate items according to the criteria previously defined. The number of factors was fixed at three, and six items were deleted according to the criteria. The first factor, accounting for 27% of the total variance, consisted of five items, the second, accounting for 21%, contained four items, and the third, accounting for 14%, consisted of three items. Cronbach's alpha coefficients for these factors were 0.87, 0.81 and 0.82, respectively, which showed adequate internal consistency. Pearson's correlations between each factor were 0.27, 0.30 and 0.55, which meant that they were satisfactorily independent of each other. Although it was difficult to interpret the meaning of each factor on the basis of the wording of the questions alone, it was hypothesized that these three factors indicate the following: Factor 1, 'sense of effort', physical dyspnoea or dysfunction of ventilation with organic cause(s) worsened on exertion; Factor 2, 'sense of anxiety', affected or amplified by psychological status; Factor 3, 'sense of discomfort', unpleasant and unrelaxed feeling at rest as well. Based on the results, a cancer dyspnoea scale containing 12 items consisting of three factors was developed. The maximum total score is 48: 20 points for 'sense of effort', 16 for 'sense of anxiety' and 12 for 'sense of discomfort'; the higher the score, the more severe the dyspnoea is (Appendix 2). An English version of the scale has been completed (Appendix 1).

### Validation phase

#### *Subjects (Table 1)*

Of the 139 outpatients and 31 inpatients who were asked to participate, two refused (1%) because of lack of time or feeling too ill, and two patients were excluded (1%) because of failure to reply. The patients' sociodemographic and clinical characteristics in this phase are shown in Table 1.

#### *Feasibility*

All patients completed the Cancer Dyspnoea Scale easily without assistance, but a few patients wavered in replying, because they felt shortness of breath on exertion, but no dyspnoea at rest. Five patients left one item unanswered, and three patients marked

**Table 2** Construct validity: factor loading pattern (followed by varimax rotation) in the validation phase ($n$ = 166)

| Item number and content | Factor 1[a] | Factor 2[b] | Factor 3[c] |
|---|---|---|---|
| 10. Narrower | 0.82 | 0.16 | –0.25 |
| 12. Stuck in the airway | 0.74 | 0.31 | 0.01 |
| 4. Short of breath | 0.69 | 0.16 | –0.27 |
| 8. Shallow | 0.63 | 0.29 | –0.26 |
| 6. Panting | 0.61 | 0.35 | –0.25 |
| 7. Breathing difficulty that one doesn't know what to do | 0.11 | 0.85 | –0.19 |
| 9. Breathing may stop | 0.25 | 0.81 | –0.15 |
| 5. Accompanied by palpitations and sweating | 0.38 | 0.67 | 0.01 |
| 11. As if drowning | 0.45 | 0.65 | –0.08 |
| 2. Exhale easily | –0.16 | –0.11 | 0.94 |
| 1. Inhale easily | –0.29 | –0.01 | 0.91 |
| 3. Breath slowly | –0.18 | –0.17 | 0.88 |

[a]'Sense of effort'; [b]'Sense of anxiety'; [c]'Sense of discomfort'.

**Table 3** Intersubscale correlation of Cancer Dyspnoea Scale factors ($n$ = 166)

| | Factor 1 | Factor 2 | Factor 3 |
|---|---|---|---|
| Factor 1 | – | – | – |
| Factor 2 | 0.65* | – | – |
| Factor 3 | 0.49* | 0.31* | – |
| Total score | 0.91* | 0.76[a] | 0.75[a] |

[a]$P$ < 0.001

double replies to an item (total eight patients; 4.8%). None of the items clearly resulted in more errors than the others. The average time required to complete the scale by the 31 inpatients was 140 s (s.d. = 44.1, median = 138).

### *Validity*

***Construct validity (Table 2)*** Since the number of factors could not be determined by the Scree test in this phase, we applied the criteria (Kaiser et al, 1960) which limit factors whose eigenvalue is greater than 1.0, according to the methodology of factor analysis (Nunnally et al, 1994). The number was fixed at 3, the same as in the development phase. Factor analysis reproduced the same loading pattern.

#### ***Intersubscale correlation (Table 3)***

There were significant correlations for all pairs of the subscale. The mean value of the intersubscale correlation coefficient was 0.48.

**Table 5** Reliability and descriptive data of the Cancer Dyspnoea Scale

| | Reliability | | Descriptive data | | |
|---|---|---|---|---|---|
| | Cronbach's alpha coefficient ($n$ = 166) | Test-retest reliability (correlation coefficient) ($n$ = 37) | Mean | (Fullscore) | s.d. ($n$ = 166) |
| Factor 1 | 0.83 | 0.71[a] | 3.8 | (20) | 3.6 |
| Factor 2 | 0.81 | 0.69[a] | 1.1 | (16) | 2.1 |
| Factor 3 | 0.94 | 0.58[a] | 3.5 | (12) | 2.7 |
| Total score | 0.64 | 0.69[a] | 8.3 | (48) | 6.9 |

[a]$P$ < 0.001.

#### ***Convergent validity (Table 4)***

Each of the factors significantly correlated with VAS of dyspnoea (average $r$ = 0.57, $P$ < 0.001) and with modified Borg's scale (average $r$ = 0.52, $P$ < 0.001). Significant correlations were also found between total score and PS, $SpO_2$, STAI, and the presence of pathophysiological cause(s).

### *Reliability (Table 5)*

***Internal consistency*** Cronbach's alpha coefficients of the subscale were 0.83, 0.81, and 0.94, respectively (average 0.86).

#### ***Test–retest reliability***

All 37 patients completed the scale the first and second time an average of 6.9 days apart (median = 7 days). Test–retest correlation coefficients between each factor and the total score were 0.71, 0.69 and 0.58 respectively ($P$ < 0.005).

### *Descriptive data (Table 5)*

Table 5 shows the mean and standard deviations for each subscale and the total score of the CDS.

### DISCUSSION

The Cancer Dyspnoea Scale, a brief self-rating questionnaire composed of 3 factors and 12 items, was developed and validated in this study using the methodology established and utilized in psychometry. To our knowledge, the CDS is the first scale that evaluates the multidimensional nature of dyspnoea. It solved the shortcomings of former assessment tools: (1) it comprises multidimensional aspects, (2) it is self-rating, (3) it is easy and simple, (4) it evaluates not physical effort evoking dyspnoea, but perceived dyspnoea itself, and (5) it has confirmed its reliability and validity in cancer patients.

**Table 4** Convergent validity: correlations between the Cancer Dyspnoea Scale and other measures

| | VAS[b] of dyspnoea | Borg's Scale | Physical status | | STAI[d] | | Pathophysiologic cause(s)[e] |
|---|---|---|---|---|---|---|---|
| | | | PS[c] | SpO2 | State | Trait | $P$ value |
| | ($n$=166) | | ($n$=135) | | ($n$=135) | | ($n$=166) |
| Factor 1[f] | 0.77[a] | 0.72[a] | 0.24[a] | –0.20 | 0.22 | 0.26[a] | <0.001 |
| Factor 2[g] | 0.53[a] | 0.41[a] | 0.18 | –0.02 | 0.28[a] | 0.33[a] | 0.119 |
| Factor 3[h] | 0.40[a] | 0.44[a] | 0.13 | –0.29[a] | 0.09 | 0.22 | <0.001 |
| Total score | 0.72[a] | 0.67[a] | 0.23[a] | –0.23[a] | 0.23[a] | 0.32[a] | <0.001 |

[a]$P$ < 0.001. [b]Visual analogue scale; [c]performance status defined by Eastern Cooperative Oncology Group; [d]State-Trait-Anxiety-Inventory; [e]evaluated by expert physician; coded: 0, absent; 1, present; [f]'Sense of effort'; [g]'Sense of anxiety'; [h]'Sense of discomfort'.

© 2000 Cancer Research Campaign 

The total- and sub-score of the CDS represent dyspnoea, which was confirmed by a significant relation to VAS and modified Borg's scale. Each sub-score of the CDS represents the different aspects of dyspnoea, which was revealed by examining the relation to physical status (PS, $SpO_2$) and psychological status (measured by STAI). Factor 2 (referred to as 'sense of anxiety') was significantly correlated with both state- and trait-anxiety, but not with $SpO_2$ or PS. These findings were interpreted as meaning that Factor 2 reflects the psychological nature of dyspnoea amplified by anxiety rather than the patient's physical condition. The findings that this factor alone was unrelated to the presence of organic causes strongly supported this interpretation. In contrast to Factor 2, Factor 1 (referred to as 'sense of effort') was significantly correlated with PS, which represents patients' gross physical status. This was interpreted as meaning that Factor 1 reflects the pathophysiological aspects of dyspnoea which are related to, and perhaps precipitated by, physical activity. On the other hand, Factor 3 (referred to as 'sense of discomfort') was significantly correlated with $SpO_2$ measured at rest. This was interpreted to mean that Factor 3 reflects an uncomfortable feeling at rest rather than shortness of breath on exertion. However, it still remains difficult to name each factor fitly. The Pearson's coefficiencies of PS, $SPO_2$ and STAI were not high, compared with that of VAS and Borg's scale. It might be explained that the scales used here for convergent validity reflected only a certain part of characteristics of dyspnoea. Further study, focusing on factors correlated with each subscore of the CDS, is needed to better understand the character of each factor.

The lack of definite independence of each factor was observed in the following findings. First, there were significant intercorrelations between each factor (average 0.48). Second, some items (such as item 11) loaded not for one, but for both of two factors. Although factor analysis reproduced the same factor loading pattern in the validations as in the development phase, construct validity was not excellent. This suggested that the multiple dimensions of dyspnoea overlap in such a complex manner and are related to each other so closely that they cannot be clearly divided into independent factors.

The CDS was confirmed to be acceptable and practicable in a clinical setting. Simplicity and ease of completion even by dyspnoeic patients is one of the most important features of the scale. However, the time required to complete this scale (average: 140 s) was longer than we expected. The difficulty of this scale, if any, may lie in confusing patients, as to which condition they should reply to, shortness of breath on exertion, or no dyspnoea at rest. Symptoms, that vary over time, such as pain, should be evaluated totally along with severity, frequency and distress (Reuben et al, 1986). Pain is, for example, often assessed at worst, at best and over the last 24 h (Daut et al, 1982). However, since the design of this scale strongly focused on brevity, it simply asked about breathing difficulty *during the past few days*, so that any dyspnoea perceived by the patient, regardless of occasion or cause, was included. Contrary to our intention, this instruction may be somewhat vague and confusing to some patients.

The limitations of this study are: (1) the sensitivity of the scale to clinical changes caused by treatment or progression of the disease over time was not validated; (2) feasibility for patients with poor PS, with severe dyspnoea was not confirmed; (3) validity for patients other than lung cancer patients was not confirmed; and (4) cross-cultural validation was also not performed. Further improvements and validation are needed.

In conclusion, the CDS developed in this study is a brief, self-rating scale that assesses the multidimensional nature of dyspnoea. Its feasibility, reliability and validity are satisfactory for clinical use, although a few problems still remain in its construction. Further study of correlated factors on the CDS might contribute to better understanding the aetiology of dyspnoea and establishing a therapeutic strategy.

### ACKNOWLEDGEMENTS

The authors gratefully acknowledge the assistance of Dr Satoshi Sasaki, MD PhD, Epidemiology and Biostatistics Division, National Cancer Center Research Institute East, in the statistical analysis. The authors acknowledge the entire staff of the Thoracic Oncology Division of National Cancer Center Hospital East and thank Yuko Kojima, RN, Kumiko Harada, RN, Yurie Sugihara, BA and Ms Miho Sakai of the Psycho-Oncology Division, National Cancer Center Research Institute East, Japan, for their research assistance. This work was supported in part by a Grant-in-Aid for Cancer Research (9-31) and the Second Term Comprehensive 10-Year Strategy for Cancer Control of the Ministry of Health and Welfare.

### REFERENCES

Ahmedzai S (1998) Palliation of respiratory symptoms. In: *Oxford Textbook of Palliative Medicine*, Doyle D, Hanks GWC and MacDonald N (eds), pp. 583–616. Oxford University Press: Oxford

American Thoracic Society (1978) Recommended respiratory disease questionnaires for use with adults and children in epidemiological research. *Am Rev Respir Dis* **118**: 7–53

Atkin RCB (1969) Measurement of feelings using visual analogue scales. *Proc R Soc Med* **62**: 989–993

Bonomi AE, Cella DF, Hahn EA, Bjordal K, Sperner-Unterweger B, Gngeri L, Bergman B, Willems-Groot J, Hanquet P and Zittoun R (1996) Multilingual translation of the Functional Assessment of Cancer Therapy (FACT) quality of life measurement system. *Qual Life Res* **5**: 309–320

Borg G (1970) Perceived exertion as an indicator of somatic stress. *Scand J Rehabil Med* **2**: 92–98

Bruera E and Ripamonti C (1998) Dyspnea in patients with Advanced cancer. In: *Principles and Practice of Supportive Oncology*. Berger A (ed). Lippincott-Raven: Philadelphia pp.295–308

Burdon JGW, Killan KJ and Jones NJ (1983) Pattern of breathing during exercise in patients with interstitial lung disease. Thorax **38**: 778–784

Burns BH and Howell JBL (1969) Disproportionaly severe breathlessness in chronic bronchitis. *Q J Med* **38**: 277–294

Catell RB (1978) The scientific use of factor analysis in behavioral and life sciences. Plenum Press: New York

Dales RE, Spitzer WO, Schechter MT and Suissa S (1989) The influence of psychological status on respiratory symptom reporting. *Am Rev Respir Dis* **139**: 1459–1463

Daut RL, Cleeland CS and Flanery RC (1983) Development of the Wisconsin Brief Pain Questionnaire to assess pain in cancer and other disease. *Pain* **17**: 197–210

Elliott MW, Adams L, Cockcroft A, Macrae KD, Murphy K and Guz A (1991) The language of breathlessness: use of verbal descriptors by Patient swith cardiopulmonary disease. *Am Rev Respir Dis* **144**: 826–832

Fletcher CM, Elmes PC, Fairbairn AS and Wood CH (1959) The significance of respiratory symptoms and the diagnosis of chronic obstructive pulmonary disease. *Qual Life Res* **2**: 257–266

Gift A (1989) Validation of a vertical visual analogue scale as a measure of clinical dyspnoea. *Rehab Nursing* **14**: 323–325

Gift A and Cahill C (1990) Psychophysiologic aspects of dyspnoea in chronic obstructive pulmonary disease: a pilot study. *Heart Lung* **19**: 252–259

Higginson I and McCarthy M (1989) Measuring symptoms in terminal cancer; are pain and dyspnoea controlled? *J R Soc Med* **82**: 264–267

Kaiser HF (1960) The application of electronic computers to factor analysis. *Educational and Psychological Measurements* **20**: 141–151

 *© 2000 Cancer Research Campaign*

McCord M and Croin-Stubbs D (1992) Operationalizing dyspnoea: focus on measurement. *Heart Lung* **21**: 167–179
McGavin CR, Artvinli M, Naoe H and McHardy GJR (1978) Dyspnoea, disability and distance walked: comparison of estimates of exercise performance in respiratory disease. *Br Med J* **2**: 241–243
Mador MJ, Rodis A and Magalang UJ (1995) Reproducibility of Borg scale measurements of dyspnoea during exercise in patients with COPD. *Chest* **107**: 1590–1597
Maler DA, Rosiello RA, Harver A, Lentine T, McGovern J and Daubenspeck J (1987) Comparison of clinical dyspnoea ratings and psychophysical measurements of respiratory sensation in obstructive airway disease. *Am Rev Respir Dis* **135**: 1229–1233
Manning HL and Schwartztstein RM (1995) Pathophysiology of dyspnoea. *N Engl J Med* **333**(**23**): 1547–1553
Medical Research Council Committee on the Aetiology of Chronic Bronchitis (1965) Standardized questionnaires on respiratory symptoms. *Br Med J* **2**: 1665
Moody L, McCormick K and Williams A (1990) Disease and symptom severity, functional status and quality of life in chronic bronchitis and emphysema (CBE). *Behav Med* **13**: 297–306
Muza SR, Silverman MT, Gilmore GC, Hellerstein HK and Kelsen SG (1990) Comparison of scales used to quantitate the sense of effort to breath in patients with chronic obstructive pulmonary disease. *Am Rev Respir Dis* **141**: 909–913
Nakazato K and Mizuguchi T (1982) Development and validation of Japanese version of State-Trait Anxiety Inventory: a study with female subjects (in Japanese). *Jpn J Psychosomatic Medicine* **22**: 107–112
Nunnally JC and Bernstein IH (1994) *Psychometric Theory*, 3rd edn, pp. 482–484. McGraw-Hill: New York
O'Connor PJ, Raglin JS and Morgan WP (1996) Psychometric correlates of perception during arm ergometry in males and females. *Int J Sports Med* **17**: 462–466
Reuben DB and Mor M (1986) Dyspnoea in terminal cancer patients. *Chest* **89**: 234–236
Ripamonti C and Bruera E (1997) Dyspnoea: pathophysiology and assessment. *J Pain Symptom Manag* **13**: 220–232
Simon PM, Shwartzstein RM, Weiss W, Lahive K, Fencl V, Teghtsoonian M and Weinberger SE (1989) Distinguishable sensations of breathlessness induced in normal volunteers. *Am Rev Respir Dis* **140**: 1021–1027
Simon PM, Shwartzstein RM, Weiss W, Fencl V, Teghtsoonian M and Weinberger SE (1990) Distinguishable types of dyspnoea in patients with shortness of breath. *Am Rev Respir Dis* **142**: 1009–1014
Spielberger CD, Gorsuch RL and Lushene RE (1970) *STAI Manual*. Consulting Psychologists Press: Palo Alto
Stoller JK, Ferranti R and Feinstein AR (1986) Further specification and evaluation of a new clinical index for dyspnoea. *Am Rev Respir Dis* **134**: 1129–1134
Wolkove N, Dajczman E, Colacone A and Kreisman H (1989) The relationship between pulmonary function and dyspnoea in obstructive lung disease. *Chest* **96**: 1247–1251

### APPENDIX 1

### The Cancer Dyspnoea Scale

We would like to ask you about your breathlessness or difficulty in breathing. Please answer each question by circling only the numbers that best describes the breathing difficulty that you felt *during the past few days*. Base your response on your first impression.

| | Not at all | A little | Somewhat | Considerably | Very much |
|---|---|---|---|---|---|
| 1 Can you inhale easily? | 1 | 2 | 3 | 4 | 5 |
| 2 Can you exhale easily? | 1 | 2 | 3 | 4 | 5 |
| 3 Can you breathe slowly? | 1 | 2 | 3 | 4 | 5 |
| 4 Do you feel short of breath? | 1 | 2 | 3 | 4 | 5 |
| 5 Do you feel breathing difficultyaccompanied by palpitations and sweating? | 1 | 2 | 3 | 4 | 5 |
| 6 Do you feel as if you are panting? | 1 | 2 | 3 | 4 | 5 |
| 7 Do you feel such breathing difficulty that you don't know what to do about it? | 1 | 2 | 3 | 4 | 5 |
| 8 Do you feel your breath is shallow? | 1 | 2 | 3 | 4 | 5 |
| 9 Do you feel your breathing may stop? | 1 | 2 | 3 | 4 | 5 |
| 10 Do you feel your airway has become narrower? | 1 | 2 | 3 | 4 | 5 |
| 11 Do you feel as if you are drowning? | 1 | 2 | 3 | 4 | 5 |
| 12 Do you feel as if something is stuckin your airway? | 1 | 2 | 3 | 4 | 5 |

### APPENDIX 2

### Calculation method

1. Add the scores for each factor together.
   Factor 1 = (items 4 + 6 + 8 + 10 + 12) – 5 = sense of effort
   Factor 2 = (items 5 + 7 + 9 + 11) – 4 = sense of anxiety
   Factor 3 = 15 – (items 1 + 2 + 3) = sense of discomfort
2. Add the total scores for each factor together = total dyspnoea

*Subtractions are to make adjustments for 0 as a state of absence of dyspnoea.

© 2000 Cancer Research Campaign
